# A web-based novel prediction model for predicting depression in elderly patients with coronary heart disease: A multicenter retrospective, propensity-score matched study

**DOI:** 10.3389/fpsyt.2022.949753

**Published:** 2022-10-18

**Authors:** Juntao Tan, Zhengguo Xu, Yuxin He, Lingqin Zhang, Shoushu Xiang, Qian Xu, Xiaomei Xu, Jun Gong, Chao Tan, Langmin Tan

**Affiliations:** ^1^Operation Management Office, Affiliated Banan Hospital of Chongqing Medical University, Chongqing, China; ^2^Department of Teaching and Research, Affiliated Banan Hospital of Chongqing Medical University, Chongqing, China; ^3^Department of Medical Administration, Affiliated Banan Hospital of Chongqing Medical University, Chongqing, China; ^4^Department of Biomedical Equipment, People’s Hospital of Chongqing Bishan District, Chongqing, China; ^5^College of Medical Informatics, Chongqing Medical University, Chongqing, China; ^6^Medical Data Science Academy, Chongqing Medical University, Chongqing, China; ^7^Library, Chongqing Medical University, Chongqing, China; ^8^Department of Gastroenterology, The Fifth People’s Hospital of Chengdu, Chengdu, China; ^9^Department of Infectious Diseases, The First Affiliated Hospital of Chongqing Medical University, Chongqing, China; ^10^Department of Information Center, The University Town Hospital of Chongqing Medical University, Chongqing, China; ^11^Department of Medical Record Management, Chongqing University Cancer Hospital, Chongqing, China; ^12^Department of Neurology, Affiliated Banan Hospital of Chongqing Medical University, Chongqing, China

**Keywords:** coronary heart disease, depression, propensity-score matching, nomogram, calculator tool

## Abstract

**Background:**

Depression is associated with an increased risk of death in patients with coronary heart disease (CHD). This study aimed to explore the factors influencing depression in elderly patients with CHD and to construct a prediction model for early identification of depression in this patient population.

**Materials and methods:**

We used propensity-score matching to identify 1,065 CHD patients aged ≥65 years from four hospitals in Chongqing between January 2015 and December 2021. The patients were divided into a training set (*n* = 880) and an external validation set (*n* = 185). Univariate logistic regression, multivariate logistic regression, and least absolute shrinkage and selection operator regression were used to determine the factors influencing depression. A nomogram based on the multivariate logistic regression model was constructed using the selected influencing factors. The discrimination, calibration, and clinical utility of the nomogram were assessed by the area under the curve (AUC) of the receiver operating characteristic curve, calibration curve, and decision curve analysis (DCA) and clinical impact curve (CIC), respectively.

**Results:**

The predictive factors in the multivariate model included the lymphocyte percentage and the blood urea nitrogen and low-density lipoprotein cholesterol levels. The AUC values of the nomogram in the training and external validation sets were 0.762 (95% CI = 0.722–0.803) and 0.679 (95% CI = 0.572–0.786), respectively. The calibration curves indicated that the nomogram had strong calibration. DCA and CIC indicated that the nomogram can be used as an effective tool in clinical practice. For the convenience of clinicians, we used the nomogram to develop a web-based calculator tool (https://cytjt007.shinyapps.io/dynnomapp_depression/).

**Conclusion:**

Reductions in the lymphocyte percentage and blood urea nitrogen and low-density lipoprotein cholesterol levels were reliable predictors of depression in elderly patients with CHD. The nomogram that we developed can help clinicians assess the risk of depression in elderly patients with CHD.

## Introduction

Depression is a common mental illness that clinically manifests as low mood, decreased willpower, and persistent fatigue (1, 2). With the development of the bio-psycho-social model and the deepening of psychological research, depression has been shown to be a risk factor for coronary heart disease (CHD) (3–5). The prevalence of depression in patients with CHD is twice that in the general population, and one in five patients with heart disease has depression (6). In addition, depression is associated with high levels of disability, especially when it occurs and persists after coronary events; it can significantly increase the incidence rate and mortality of heart disease (7). Furthermore, the mutually reinforcing bidirectional association between depression and CHD is clear (8–10).

Despite the availability of various treatment methods for depression, the treatment effects remain poor due to the lack of medical resources and difficulties in early detection of depression (11). As a result, a risk prediction model for depression is essential to facilitate early detection of this disease. In recent years, more studies have aimed to construct prediction models for early screening of depression (12–14). Biological parameters such as triglyceride level, white blood cell count, and protein have been confirmed to be important influencing factors of depression prediction models (15, 16). Although prediction models have been applied to predict the risk of depression in different populations, it remainsl underused in CHD population. In particular, there is a lack of prediction model that assesses the risk of developing depression in elderly patients with CHD.

Therefore, the purpose of this study is to identify the independent factors affecting depression in elderly patients with CHD from the general demographic characteristics, comorbidities, and laboratory indicators because these indicators are affordable and easy to obtain. At the same time, a prediction model is constructed to help clinicians determine the probability of depression in elderly patients with CHD and take intervention measures to minimize the possibility of depression.

## Materials and methods

### Data source

The study data were obtained from electronic medical records of four tertiary hospitals on the Big Data Platform of Medical Data Research Institute of Chongqing Medical University. At present, the platform has collected more than 40 million electronic medical records from seven tertiary hospitals of Chongqing, and anonymized all information related to patient privacy. A total of 50 patient records were collected from the Yongchuan Hospital of Chongqing Medical University, 360 from the Second Affiliated Hospital of Chongqing Medical University, and 470 from the University-Town Hospital of Chongqing Medical University. Patients from above the three centers were divided into the training. The patient records collected from the Third Affiliated Hospital of Chongqing Medical University (*n* = 185) were used as the external validation set.

The Ethics Committee of the Affiliated Banan Hospital of Chongqing Medical University approved the study. Written informed consent for participation was not required for this study due to its retrospective design, and the study was undertaken in accordance with national legislation and institutional requirements.

### Definition

The study targeted patients who were initially diagnosed with CHD, which was later confirmed by clinical, biochemical, and imaging data or past medical records; the diagnosis was in accordance with the “Chinese guidelines on the management of coronary heart disease” (17). The types of CHD considered in the inclusion criteria were angina pectoris, myocardial infarction, occult CHD, and ischemic cardiomyopathy. Depression was jointly determined by two psychiatrists trained in consistent procedures according to Hamilton Depression Rating Scale during the patient’s hospitalization (18). Before assessment, the patients were informed of the guidance, emphasizing that the assessment time was the actual feeling in the current or the past week. A score of 7 or higher indicates the presence of depression. In addition, all patients in this study had been diagnosed with CHD before depression was identified.

### Inclusion and exclusion criteria

The inclusion criteria were as follows: (i) data obtained from 2015 to 2021, (ii) patients aged ≥65 years, and (iii) hospitalization(s) with CHD. The exclusion criteria were as follows: (i) patients with cancer or other serious complications, (ii) patients with hospital stay ≤1 day, and (iii) patients with >30% missing data. The study selection process is depicted in the flow chart (see Supplementary Figure 1 for details).

### Data collection

In this study, patients were divided into two groups depending on the presence of comorbid depression: the depression group and the non-depression group. For all patients, we collected clinical data, including information related to hypertension, diabetes, hyperlipidemia, chronic gastritis, pulmonary infection, atrial fibrillation, cardiac insufficiency, smoking status, alcohol consumption status, sex, and age as well as data related to neutrophilic granulocyte percentage (NEUT%), lymphocyte percentage (LYM%), monocyte percentage (MO%), red blood cell (RBC) count, white blood cell (WBC) count, platelet count, and the levels of γ-glutamyltransferase (GGT), creatinine (CREA), blood potassium, total bilirubin (TBIL), blood calcium, uric acid (UA), blood urea nitrogen (BUN), total protein (TP), alkaline phosphatase (ALP), low-density lipoprotein-cholesterol (LDL-C), hemoglobin (HGB), alanine aminotransferase (ALT), blood glucose (BG), aspartate aminotransferase (AST), triglycerides (TGs), total cholesterol (TC), and high-density lipoprotein-cholesterol (HDL-C).

### Statistical analyses

For this study, statistical analyses were performed using SPSS 22.0 and R (version 4.0.2, Vienna, Austria). Propensity-score matching (PSM) analysis was performed with the nearest neighbor method for data matching (ratio, 4; caliper, 0.04) (19). Univariate logistic regression analyses and least absolute shrinkage and selection operator (LASSO) regression analyses were used to identify independent factors (20). A nomogram was constructed on the basis of these independent factors. The area under the curve (AUC) of the receiver operating characteristic (ROC) curve was determined to evaluate the accuracy of the nomogram (21). Calibration curves were established to evaluate the calibration of the nomogram (22). In addition, decision curve analysis (DCA) and clinical impact curve (CIC) analysis were performed to demonstrate the clinical benefit of the nomogram (23, 24). The multiple imputation method was used to fill in the missing continuous variables (25). The enumeration data were expressed by rate and percentage, and the chi-square test was used for comparisons between groups. The measurement data were represented by median and interquartile range [M(Q25-Q75)], and intergroup comparisons were performed by the Mann–Whitney U test. All statistical analyses were performed using two-sided tests, and *P* < 0.05 was considered statistically significant.

## Results

### Patient characteristics

A total of 1,065 elderly patients with CHD were included in this study, including 215 patients with depression and 850 patients without depression. They were divided into the training set (depression [*n* = 176]; non-depression [*n* = 704]) and external validation set (depression [*n* = 37]; non-depression [*n* = 148]). The training set showed no significant difference between the two groups after PSM based on center, age, sex, smoking status, and alcohol consumption status (Table 1). The external validation set also showed no significant difference between the two groups after PSM analysis (Supplementary Table 1). The Mann–Whitney U test showed no significant difference in all missing variables before and after multiple imputation in the training set (Table 2), as well as the external validation set (Supplementary Table 2).

**TABLE 1:** The baseline data with propensity-score matching analysis in the training set.

Variables	Training set (*N* = 880)		*P*-values
		
	Depression (*n* = 176)	Non-depression (*n* = 704)	
Center, n (%)			1.000
A	10 (20.00)	40 (80.00)	
B	72 (20.00)	288(80.00)	
C	94 (20.00)	376(80.00)	
Age (IQR, year)	75.00 (70.00, 80.00)	75.00 (70.00, 80.00)	0.894
Sex, n (%)			0.773
Male	58 (32.95)	224 (31.82)	
Female	118 (67.05)	480 (68.18)	
Smoking status, n (%)			0.899
Yes	34 (19.32)	139 (19.74)	
No	142 (80.68)	565 (80.26)	
Drinking status, n (%)			0.891
Yes	29 (16.48)	113 (16.05)	
No	147 (83.52)	591 (83.95)	

IQR, interquartile range.

**TABLE 2:** Comparison of continuous variables in the training set before and after multiple imputation.

Variables	Training set (*N* = 880)		*P*-values
		
	Before interpolation	After interpolation	
TBIL (IQR, umol/l)	10.85 (8.00, 14.59)	10.86 (8.00, 14.64)	0.880
TP (IQR, g/L)	67.45 (63.10, 72.44)	67.35 (63.02, 72.41)	0.872
ALT (IQR, IU/L)	17.00 (12.00, 25.00)	17.00 (12.00, 25.00)	0.872
AST (IQR, IU/L)	20.99 (17.02, 27.31)	20.96 (17.02, 27.31)	0.998
GGT (IQR, IU/L)	25.42 (17.77, 42.00)	25.00 (17.00, 42.01)	0.799
ALP (IQR, IU/L)	77.25 (62.90, 93.05)	77.90 (63.00, 93.03)	0.907
Platelet count (IQR, × 10^9^/L)	181.00 (138.00, 222.00)	182.00 (138.75, 222.00)	0.932
MO% (IQR,%)	6.30 (4.90, 8.10)	6.30 (4.90, 8.10)	0.987
CREA (IQR, umol/l)	72.76 (59.14, 93.89)	72.78 (59.52, 93.93)	0.921
BUN (IQR, mmol/L)	6.35 (4.96, 8.54)	6.35 (4.96, 8.57)	0.950
UA (IQR, umol/L)	335.03 (264.96, 412.15)	336.30 (266.05, 412.28)	0.875
Blood potassium (IQR, mmol/L)	4.05 (3.76, 4.33)	4.05 (3.76, 4.32)	0.853
Blood calcium (IQR, mmol/L)	2.27 (2.16, 2.38)	2.27 (2.16, 2.38)	0.831
BG (IQR, mmol/L)	5.94 (5.19, 7.39)	5.92 (5.21, 7.29)	0.825
TGs (IQR, mmol/L)	1.22 (0.93, 1.77)	1.23 (0.93, 1.76)	0.946
TC (IQR, mmol/L)	4.27 (3.45, 5.06)	4.26 (3.45, 5.05)	0.873
HDL-C (IQR, mmol/L)	1.20 (1.00, 1.44)	1.20 (0.98, 1.44)	0.633
LDL-C (IQR, mmol/L)	2.36 (1.73, 3.03)	2.36 (1.71, 3.04)	0.961

TBIL, total bilirubin; TP, total protein; ALT, alanine aminotransferase; AST, aspartate aminotransferase; GGT, γ-glutamyltransferase; ALP, alkaline phosphatase; MO%, monocyte percentage; CREA, creatinine; BUN, blood urea nitrogen; UA, uric acid; BG, blood glucose; TGs, triglycerides; TC, total cholesterol; HDL-C, high-density lipoprotein-cholesterol; LDL-C, low-density lipoprotein-cholesterol; IQR, interquartile range.

### Selection of predictors for depression in elderly patients with coronary heart disease

**FIGURE 1:**
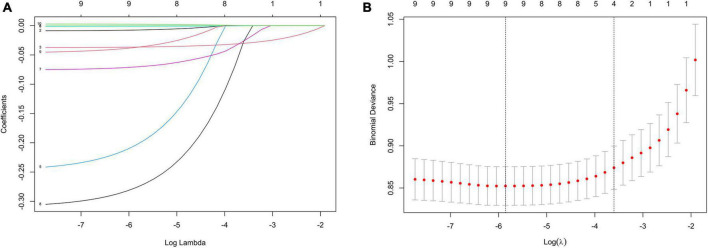
Feature selection by LASSO. **(A)** LASSO coefficient profiles (*y*-axis) of the nine features. The upper x-axis shows the average numbers of predictors and the lower *x*-axis shows the log(λ). **(B)** Ten-fold cross-validation for tuning parameter selection in the LASSO model.

Multicollinearity of the independent variables was checked by calculating the Variance Inflation Factor (VIF) (26). If VIF < 10, there is no multicollinearity between explanatory variables. If 10 ≤ VIF ≤ 20, there is a certain amount of autocorrelation between explanatory variables. If VIF > 20, there is serious multicollinearity between explanatory variables (27). All variables included in this study are having VIF < 10, which avoid the influence caused by the interdependence relationships between variables (Supplementary Table 3). The training set showed significant differences in baseline data in the comparison between the depression and non-depression groups, including NEUT% (*P* < 0.001), LYM% (*P* < 0.001), platelet count (*P* = 0.003), and CREA (*P* = 0.003), blood potassium (*P* = 0.004), UA (*P* < 0.001), BUN (*P* < 0.001), LDL-C (*P* = 0.019), and BG (*P* = 0.003) levels (Table 3). LASSO regression analysis and multivariate logistic regression analysis identified three variables discriminating depression from non-depression (Figure 1 and Table 4): LYM% (OR = 0.962, 95% CI = 0.955–0.969), BUN level (OR = 0.882, 95% CI = 0.827–0.941), and LDL-C level (OR = 0.749, 95% CI = 0.610–0.921).

**TABLE 3:** Univariate analyses of variables associated with depression.

Variables	Depression (*n* = 176)	Non-depression (*n* = 704)	*P*-values
**Comorbidities**			
Hypertension (n,%)	113 (64.20)	467 (66.34)	0.594
Diabetes (n,%)	59 (33.52)	198 (28.13)	0.159
Hyperlipidemia (n,%)	14 (7.95)	76 (10.80)	0.266
Chronic gastritis (n,%)	21 (11.93)	74 (10.51)	0.587
Pulmonary infection (n,%)	25 (14.20)	101 (14.37)	0.962
Atrial fibrillation (n,%)	28 (15.91)	98 (13.92)	0.501
Cardiac insufficiency (n,%)	68 (38.64)	316 (44.89)	0.135
**Laboratory indicators**			
TBIL (IQR, umol/l)	10.18 (7.88, 13.48)	11.08 (8.00, 14.90)	0.172
TP (IQR, g/L)	67.19 (63.44, 72.22)	67.44 (62.88, 72.50)	0.919
ALT (IQR, IU/L)	17.00 (13.00, 23.55)	17.00 (12.00, 25.21)	0.799
AST (IQR, IU/L)	20.00 (17.28, 25.00)	21.00 (17.00, 28.00)	0.157
GGT (IQR, IU/L)	24.31 (17.83, 36.13)	25.15 (17.00, 45.13)	0.373
ALP (IQR, IU/L)	76.63 (62.95, 93.03)	78.00 (63.00, 93.03)	0.703
RBC (IQR, × 10^12^/L)	4.17 (3.74, 4.48)	4.14 (3.79, 4.49)	0.722
HGB (IQR, g/L)	125.50 (113.75, 133.25)	125.00 (115.00, 135.00)	0.744
WBC (IQR, × 10^9^/L)	6.21 (5.31, 7.69)	6.37 (5.20, 8.21)	0.404
Platelet count (IQR, × 10^9^/L)	198.5 (148.00, 233.75)	179.00 (136.75, 220.00)	0.003
NEUT% (IQR,%)	66.65 (59.60, 75.30)	70.70 (63.30, 79.11)	<0.001
LYM% (IQR,%)	34.05 (23.05, 66.13)	68.50 (58.20, 77.53)	<0.001
MO% (IQR,%)	6.35 (5.38, 8.10)	6.30 (4.80, 8.10)	0.268
CREA (IQR, umol/l)	68.35 (56.13, 84.83)	74.00 (60.08, 96.68)	0.003
BUN (IQR, mmol/L)	5.77 (4.66, 7.29)	6.57 (5.06, 8.96)	<0.001
UA(IQR, umol/L)	303.54 (244.31, 383.64)	342.15 (273.68, 418.34)	<0.001
Blood potassium (IQR, mmol/L)	3.98 (3.72, 4.18)	4.07 (3.77, 4.36)	0.004
Blood calcium (IQR, mmol/L)	2.27 (2.15, 2.36)	2.27 (2.16, 2.39)	0.268
BG (IQR, mmol/L)	5.57 (5.06, 6.73)	6.03 (5.25, 7.46)	0.003
TGs (IQR, mmol/L)	1.18 (0.90, 1.71)	1.25 (0.94, 1.77)	0.333
TC (IQR, mmol/L)	4.25 (3.31, 4.87)	4.26 (3.50, 5.08)	0.250
HDL-C (IQR, mmol/L)	1.20 (1.01, 1.51)	1.19 (0.96, 1.44)	0.177
LDL-C (IQR, mmol/L)	2.18 (1.65, 2.81)	2.40 (1.74, 3.07)	0.019

TBIL, total bilirubin; TP, total protein; ALT, alanine aminotransferase; AST, aspartate aminotransferase; GGT:γ-glutamyltransferase; ALP, alkaline phosphatase; RBC, red blood cells; HGB, hemoglobin; WBC, white blood cells; NEUT%:neutrophilic granulocyte percentage; LYM%, lymphocyte percentage; MO%, monocyte percentage; CREA, creatinine; BUN, blood urea nitrogen; UA, uric acid; BG, blood glucose; TGs, triglycerides; TC, total cholesterol; HDL-C, high-density lipoprotein-cholesterol; LDL-C, low-density lipoprotein-cholesterol; IQR, interquartile range.

**TABLE 4:** Results of multivariate logistic regression model.

Variables	β	SE	OR (95%CI)	*P*-values
LYM%	−0.039	0.004	0.962(0.955–0.969)	<0.001
BUN	−0.125	0.033	0.882(0.827–0.941)	<0.001
LDL-C	−0.288	0.105	0.749(0.610–0.921)	0.006

LYM%, lymphocyte percentage; BUN, blood urea nitrogen; LDL-C, low-density lipoprotein-cholesterol; SE, standard error; OR, odds ratio; CI, confidence interval.

### Nomogram construction and performance

**FIGURE 2:**
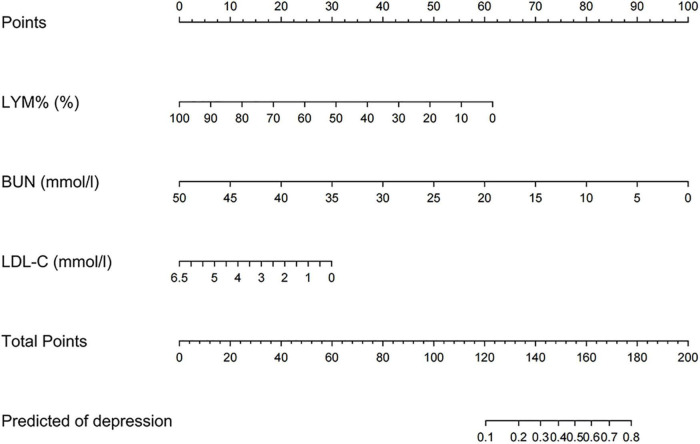
Nomogram predicting depression in patients with coronary heart disease (CHD).

**FIGURE 3:**
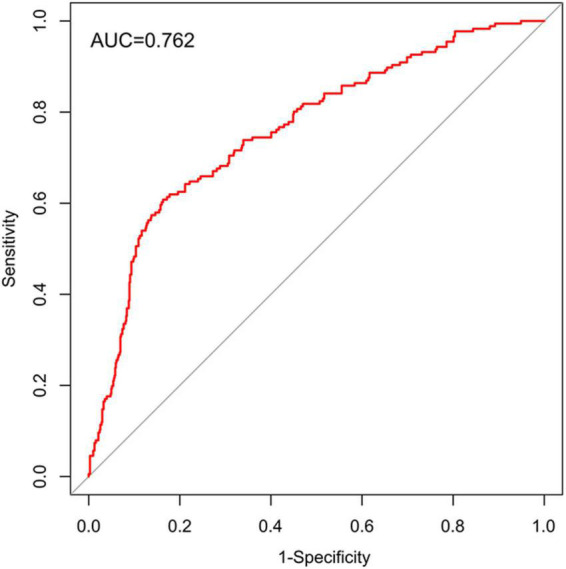
Area under the curve (AUC) of the ROC curve in the training set.

**FIGURE 4:**
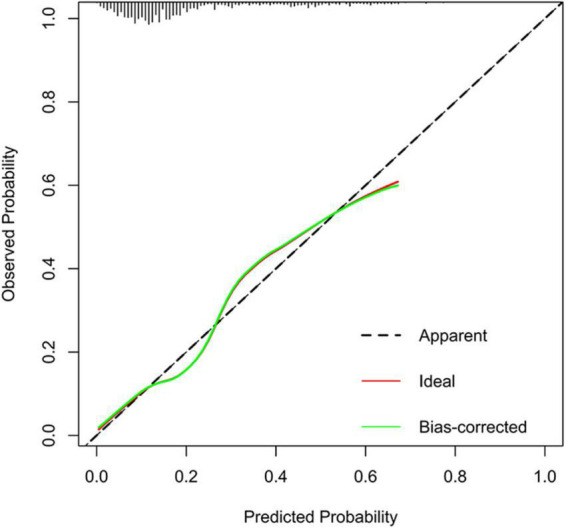
Calibration curves of the depression incidence risk nomogram prediction in training set.

On the basis of the logistic regression model, a nomogram was constructed to predict depression in elderly patients with CHD. To use the nomogram, the endpoints of line segments corresponding to each of the patient’s risk indicators were selected and vertical lines were made to the scoring standard axis, with the point of intersection serving as the scoring value of the single indicator. After summing the scores of each index, the total score was determined, the corresponding score point on the total score axis was identified, and a vertical line was drawn to the risk axis. The intersection represented the risk of depression for the patient (Figure 2). An AUC of 0.762 (95% CI = 0.722–0.803) was calculated as the accuracy for this nomogram, indicating that the nomogram model had good prediction accuracy (Figure 3). An AUC of 0.679 (95% CI = 0.572–0.786) was calculated in the external validation set. The details for the external validation set are shown in Supplementary Figure 2. Moreover, the calibration curves (bootstraps = 1,000) of the training set indicated that the nomogram had strong calibration (Figure 4). The details of the external validation set are shown in Supplementary Figure 3.

### Clinical utility of the nomogram

**FIGURE 5:**
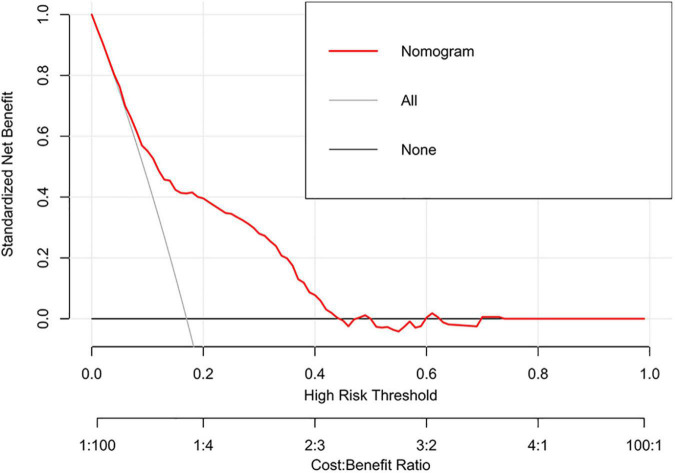
Decision curve analysis (DCA) of the nomogram in the training set.

**FIGURE 6:**
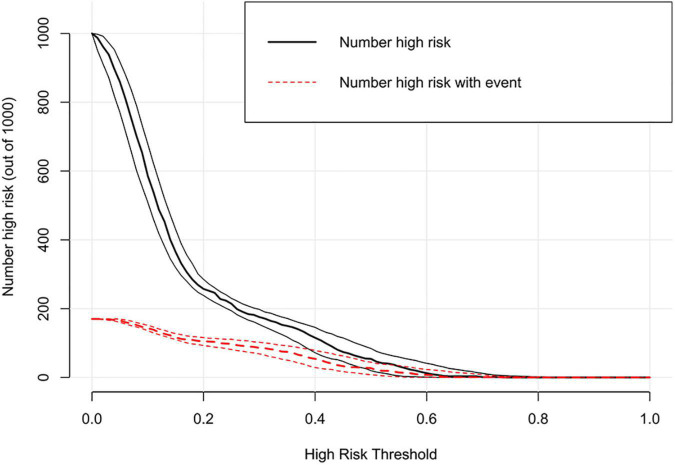
Clinical impact curve of the nomogram in the training set.

Decision curve analysis showed that the range of net benefit in this nomogram was between 0.09 and 0.43 (Figure 5). The details of the external validation set are shown in Supplementary Figure 4. In addition, the CIC is further drawn according to DCA to evaluate the clinical influence of nomogram, so as to intuitively present its substantive value (Figure 6). In the training set, the CIC shows that the nomogram has significant predictive power. When the risk threshold exceeded 18%, the estimated number of patients with depression was closer to the real number of patients with depression. The details of the external validation set are shown in Supplementary Figure 5.

### Construction of a web app to facilitate easy access to the nomogram

**FIGURE 7:**
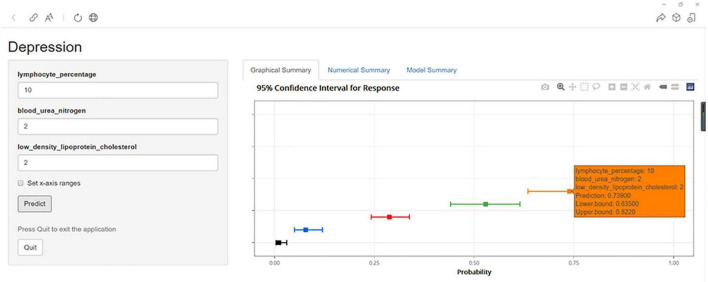
An example of depression prediction using the nomogram in patients with CHD *via* a link.

To facilitate the usage of the nomogram by clinicians, we built an operation interface on a webpage^1^ to calculate the exact probability of depression. For example, when a patient has an LYM% of 10.00%, BUN level of 2.00 mmol/L, and LDL-C level of 2.00 mmol/L, the probability of depression would be 0.739 (95% CI = 0.635–0.822) (Figure 7).

## Discussion

Depression seriously threatens the quality of life of patients with CHD. Thus, early detection of the risk of depression in patients with CHD is helpful in improving their quality of life. The depression prediction model developed in this study can help clinicians identify high-risk groups early and formulate personalized preventive measures. The clinical efficacy of the model was evaluated in terms of discrimination, calibration, and clinical utility through AUC measurements, calibration curve evaluations, and DCA and CIC, respectively. The results showed that the overall effectiveness of the prediction model is good, and that the web-based calculator tool can make it more convenient for clinicians to use.

Our study showed that a low lymphocyte percentage was a risk factor for depression in elderly patients with CHD. The lymphocyte count is one of the most important peripheral blood inflammatory indices (28). Several studies have shown that peripheral blood inflammatory indices are closely related to depression (29–31). These inflammatory markers (such as the neutrophil/lymphocyte, platelet/lymphocyte, and monocyte/lymphocyte ratios) have also been examined in relation to the severity of depression or suicidality in major depressive disorder and other mood disorders (32, 33). Puangsri et al. reported that suicidal attempts in patients with major depressive disorders were associated with a lower lymphocyte percentage as well as elevated monocyte counts and monocyte/lymphocyte ratios (34). For lymphocytes, they are the main effectors of adaptive immunity which facilitate pathogen-specific immune recognition, immune memory production, and regulation of host immune homeostasis (35). Lymphocytes perform a regulatory or protective function in adaptive immunity and reduced lymphocytes indicate poor general condition and physiological stress (36, 37). Therefore, lymphocyte percentage should be regularly monitored in elderly patients with CHD in clinical practice, which will help clinicians obtain an appropriate understanding of the risk of depression and disease progression.

Low blood urea nitrogen levels have also been previously reported in major depressive disorder patients (38). Lee reported that in comparison with a control group, the depression group was more likely to show higher triglyceride levels and tended to have lower hematocrit values and blood urea nitrogen levels (39). Consistent with this finding, decreased blood urea nitrogen levels were demonstrated to be associated with depression in our study. Low blood urea nitrogen levels in elderly patients with CHD may be attributed to insufficient intake of protein-rich foods such as fish, meat and eggs, resulting in a negative nitrogen balance and reduced concentrations of blood urea nitrogen, a metabolite of protein. A large number of studies have confirmed that insufficient protein intake will lead to malnutrition (40–43). Meanwhile, a recent study showed that depression and malnutrition were found to be risk factors for adverse outcomes in patients with CHD (44). Among the 547 CHD patients included in the study, a total of 20.6% of the participants were found to have comorbidities of depression and malnutrition; both moderate to severe depression (adjusted hazard ratio [HR], 1.674; 95%CI, 1.098–2.551) and moderate to severe malnutrition (adjusted HR, 1.686; 95%CI, 1.073–2.648) were observed to be risk factors for the composite end point. Therefore, clinicians should pay close attention to changes in the blood urea nitrogen levels in elderly patients with CHD, and that the risk of depression should be especially considered when these patients show a reduction in blood urea nitrogen levels.

Our study also showed that a decreased low-density lipoprotein-cholesterol level was a risk factor for depression in elderly patients with CHD, which was consistent with the conclusions of a meta-analysis published in 2016 (45). This meta-analysis included 36 different studies and found an overall association between low-density lipoprotein-cholesterol levels and depression (mean difference, −4.29; 95% CI = −8.19, −0.40; *P* = 0.03). A study of the association of serum cholesterol and its fractions with depression disorder and suicide attempts in 467 adult participants in the Mexican mestizo population showed that lower cholesterol levels were significantly associated with major depressive disorder and suicide attempt after adjusting for covariates (46). Some studies further investigated the link between low cholesterol levels and depression, and the results also showed that low cholesterol levels were associated with a higher risk of depression (47–49). Aijänseppä et al. examined the relationship between depression and low-density lipoprotein-cholesterol and high-density lipoprotein-cholesterol levels in men aged 70–89 years over a 30-year follow-up interval across seven countries and reported a lower mean serum low-density lipoprotein-cholesterol level in the depressed group (50). Together, some studies provide evidence suggesting that the mechanism guiding the relationship between low-density lipoprotein-cholesterol and depression pathogenesis may involve cholesterol depletion-mediated alterations in central nerve terminal structure and function in turn altering neurotransmitter uptake and release, including serotonin, ultimately leading to depression (51–53).

In addition, some other indicators have also been confirmed to be risk factors for depression in patients with CHD. For example, the results of a study including 3,237 patients showed that young women had a higher rate of depression than men and older women of the same age, and female patients aged <55 years were more prone to depression (54). Another study also showed that women, diabetes, and higher Gensini score were independent risk factors for depression in patients with CHD (55). Ming Chen et al. found that poor sleep quality was associated with both anxiety and depression symptoms in Chinese patients with CHD (56).

Studies describing prediction models of depression in elderly patients with CHD are limited. The advantage of the nomogram constructed in this study is that all predictors can be easily obtained from regular examinations. Moreover, to simplify the clinical application of this model, we have built an operation interface on the web page, which will facilitate informed decision-making regarding prophylactic treatments for depression.

However, this study also had several limitations. First, this was a retrospective study, and sample selection bias was inevitable. However, a multicenter and relatively large training set was used to build the models which were further subjected to external validation. Second, considering the extreme unbalance between the sample size of the depression group and that of the non-depression group, we performed PSM to adjust for patient differences between the depression and non-depression groups. Even so, there may still be inevitable differences between the two groups. Third, some potential influencing factors were ignored due to the high percentage of missing values. The addition of these factors may improve the prediction efficiency of the model. Besides, the efficacy of the predictors of depression selected in this study also needs further validation. Therefore, in the future, we will enable prospective studies with more detailed data and studies with larger sample sizes to verify or update our current research results.

## Conclusion

In conclusion, this study demonstrated that lymphocyte percentage, blood urea nitrogen level, and low-density lipoprotein cholesterol level were associated with depression. The nomogram constructed from this study may be a promising tool for predicting depression in elderly patients with coronary heart disease.

## Data availability statement

The raw data supporting the conclusions of this article will be made available by the authors, without undue reservation.

## Ethics statement

The Ethics Committee of the Affiliated Banan Hospital of Chongqing Medical University approved the study. Written informed consent for participation was not required for this study due to its retrospective design, and the study was undertaken in accordance with national legislation and institutional requirements.

## Author contributions

JT and LT designed the study. JT, ZX, QX, LZ, and XX collected and organized the data. JT, SX, JG, CT, and YH analyzed the data. JT drafted the manuscript. LT contributed to the critical revision of the manuscript. All authors contributed to the final manuscript and approved the submitted version.

## Footnotes

1
https://cytjt007.shinyapps.io/dynnomapp_depression/
