# Supplementary material for: A web-based novel prediction model for predicting depression in elderly patients with coronary heart disease: A multicenter retrospective, propensity-score matched study
Source: Front Psychiatry. 2022 Oct 18;13:949753. doi: 10.3389/fpsyt.2022.949753 (PMC9624295; doi:10.3389/fpsyt.2022.949753)
Supplement: Supplementary file 1 [file Data_Sheet_1.docx]

**Supplementary information**

**Figure S1 Flow of inclusions and exclusions**

**
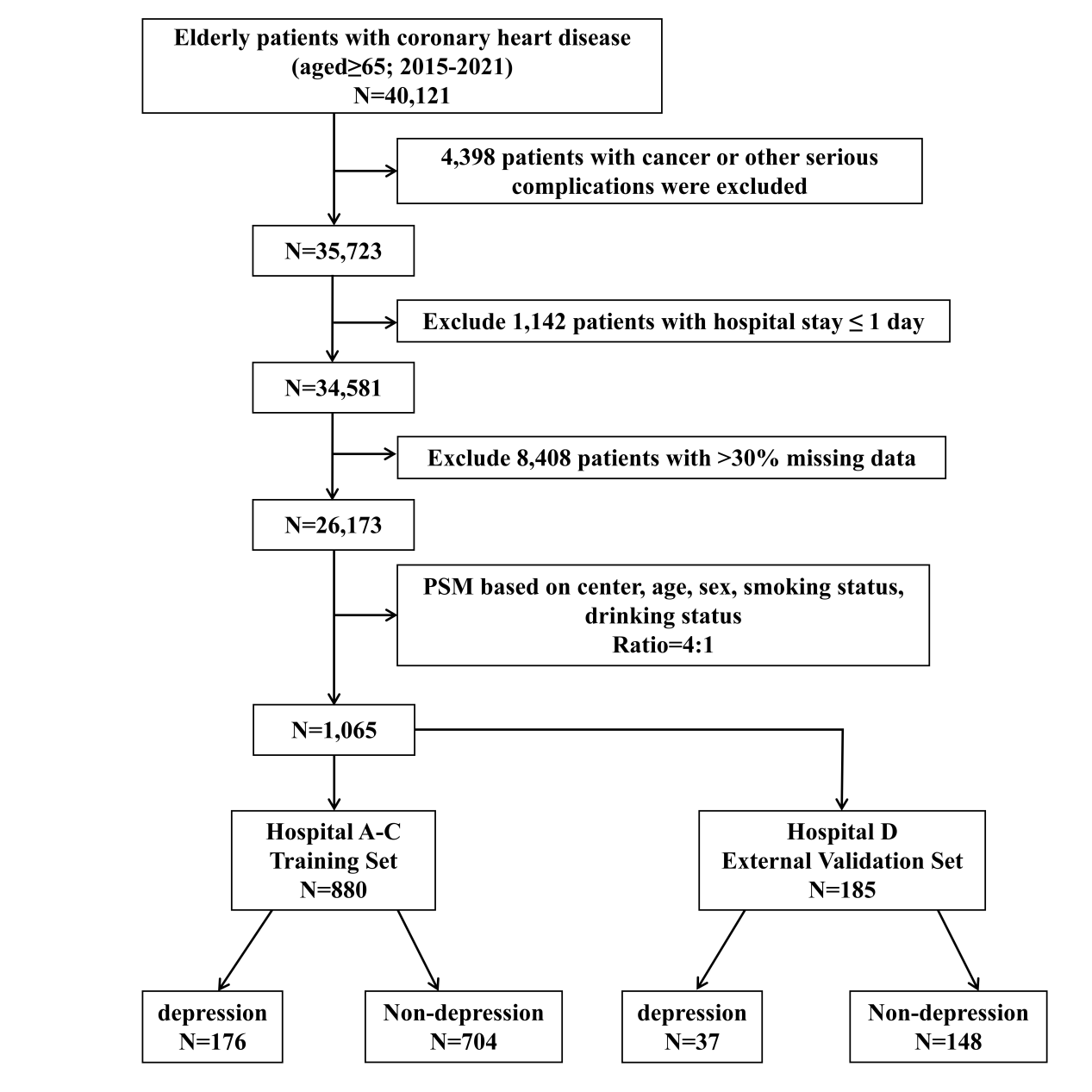
**

**Table S1 The baseline data with propensity-score matching analysis in the external validation set**

| Variables | External Validation Set (N=185) | | *P* values |
| --- | --- | --- | --- |
|  | depression (n=37) | Non-depression (n=148) |  |
| Age (IQR, year) | 71.00 (69.00, 79.00) | 75.00 (70.00, 80.00) | 1.000 |
| Sex, n (%) |  |  | 1.000 |
| Male | 11 (29.73) | 44(29.73) |  |
| Female | 26 (70.27) | 104(70.27) |  |
| Smoking status, n (%) |  |  | 1.000 |
| Yes | 8 (21.62) | 32 (21.62) |  |
| No | 29 (78.38) | 116 (78.38) |  |
| Drinking status, n (%) |  |  | 1.000 |
| Yes | 6 (16.22) | 24 (16.22) |  |
| No | 31 (83.78) | 124 (83.78) |  |

*IQR: interquartile range.*

**Table S2 Comparison of continuous variables in the training set before and after multiple imputation in the external validation set**

| Variables | External Validation Set (N=185) | | *P* values |
| --- | --- | --- | --- |
|  | Before interpolation | After interpolation |  |
| GGT (IQR, IU/L) | 24.30 (17.00, 42.00) | 24.40 (17.00, 42.00) | 0.984 |
| ALP (IQR, IU/L) | 73.85 (61.00, 98.55) | 74.60 (61.00, 94.10) | 0.991 |
| platelet count (IQR, ×109/L) | 182.50 (140.00, 237.75) | 183.00 (140.00, 237.00) | 0.996 |
| BUN (IQR, mmol/L) | 6.01 (4.71, 7.47) | 6.03 (4.71, 7.49) | 0.928 |
| UA (IQR, umol/L) | 320.00 (267.05, 402.85) | 320.00 (267.40, 403.00) | 0.936 |
| blood potassium (IQR, mmol/L) | 3.93 (3.71, 4.26) | 3.93 (3.67, 4.26) | 0.945 |
| blood calcium (IQR, mmol/L) | 2.11 (1.97, 2.22) | 2.15 (2.00, 2.25) | 0.134 |
| BG (IQR, mmol/L) | 5.65 (5.14, 7.68) | 5.78 (5.13, 7.68) | 0.938 |
| TGs (IQR, mmol/L) | 1.30 (0.98, 1.87) | 1.27 (0.95, 1.72) | 0.502 |
| TC (IQR, mmol/L) | 4.35 (3.60, 5.13) | 4.21 (3.58, 5.03) | 0.407 |
| HDL-C (IQR, mmol/L) | 1.25 (1.08, 1.51) | 1.20 (1.00, 1.41) | 0.235 |
| LDL-C (IQR, mmol/L) | 2.32 (1.84, 2.91) | 2.32 (1.81, 2.88) | 0.797 |

*GGT:γ-glutamyltransferase; ALP: alkaline phosphatase; BUN: blood urea nitrogen; UA: uric acid; BG: blood glucose; TGs: triglycerides; TC: total cholesterol; HDL-C: high-density lipoprotein-cholesterol; LDL-C: low-density lipoprotein-cholesterol; IQR: interquartile range.*

**Table S3 Values of variance Inflation Factor in all independent variables**

| Variables | VIF |
| --- | --- |
| hypertension | 1.128 |
| diabetes | 1.346 |
| hyperlipidemia | 1.148 |
| chronic gastritis | 1.096 |
| pulmonary infection | 1.236 |
| atrial fibrillation | 1.301 |
| cardiac insufficiency | 1.232 |
| total bilirubin | 1.227 |
| total protein | 1.381 |
| alanine aminotransferase | 3.153 |
| aspartate aminotransferase | 3.060 |
| γ-glutamyltransferase | 1.404 |
| alkaline phosphatase | 1.220 |
| red blood cells | 3.296 |
| hemoglobin | 3.515 |
| white blood cells | 1.602 |
| platelet count | 1.214 |
| neutrophilic granulocyte percentage | 1.670 |
| lymphocyte percentage | 1.144 |
| monocyte percentage | 1.375 |
| creatinine | 1.132 |
| blood urea nitrogen | 1.677 |
| uric acid | 1.419 |
| blood potassium | 1.180 |
| blood calcium | 1.136 |
| blood glucose | 1.314 |
| triglycerides | 2.025 |
| total cholesterol | 9.324 |
| high-density lipoprotein-cholesterol | 2.711 |
| low-density lipoprotein-cholesterol | 9.523 |

*VIF：VarianceInflation Factor.*

**
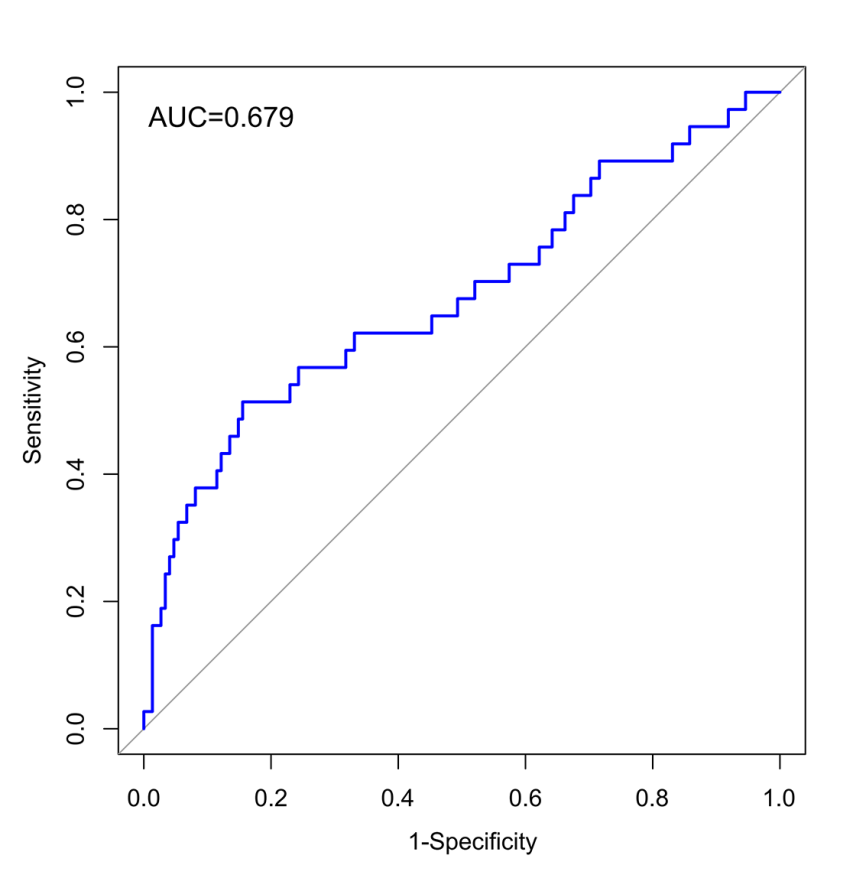
**

**Figure S2 AUC of the ROC curve in the external validation set.**

**
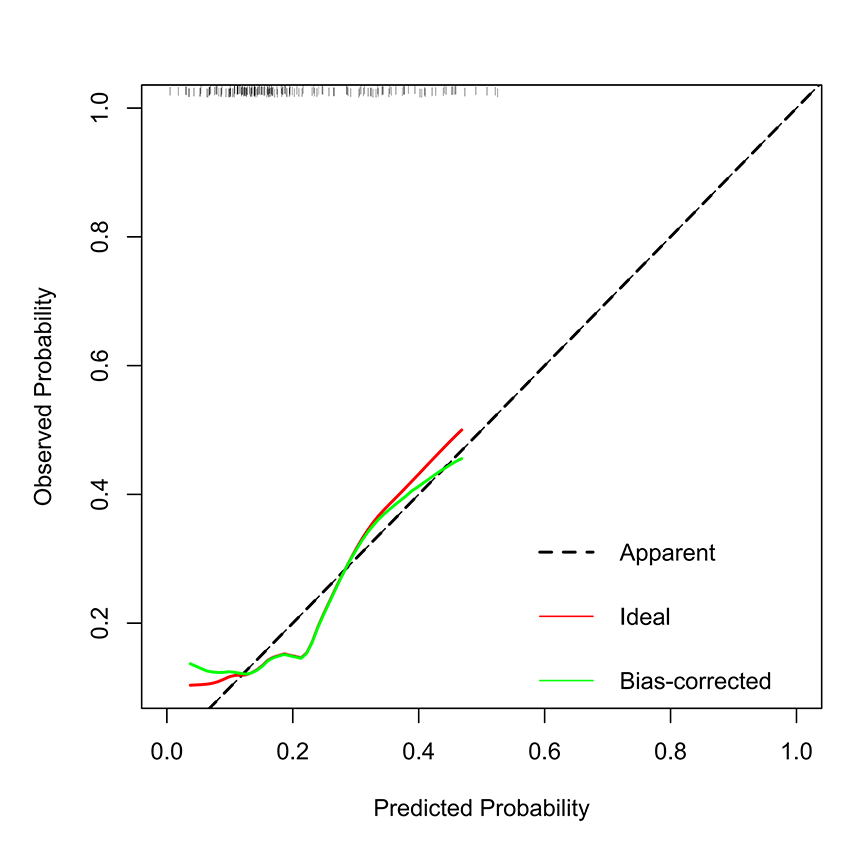
**

**Figure S3 Calibration curves of the depression incidence risk nomogram prediction in the external validation set.**

**
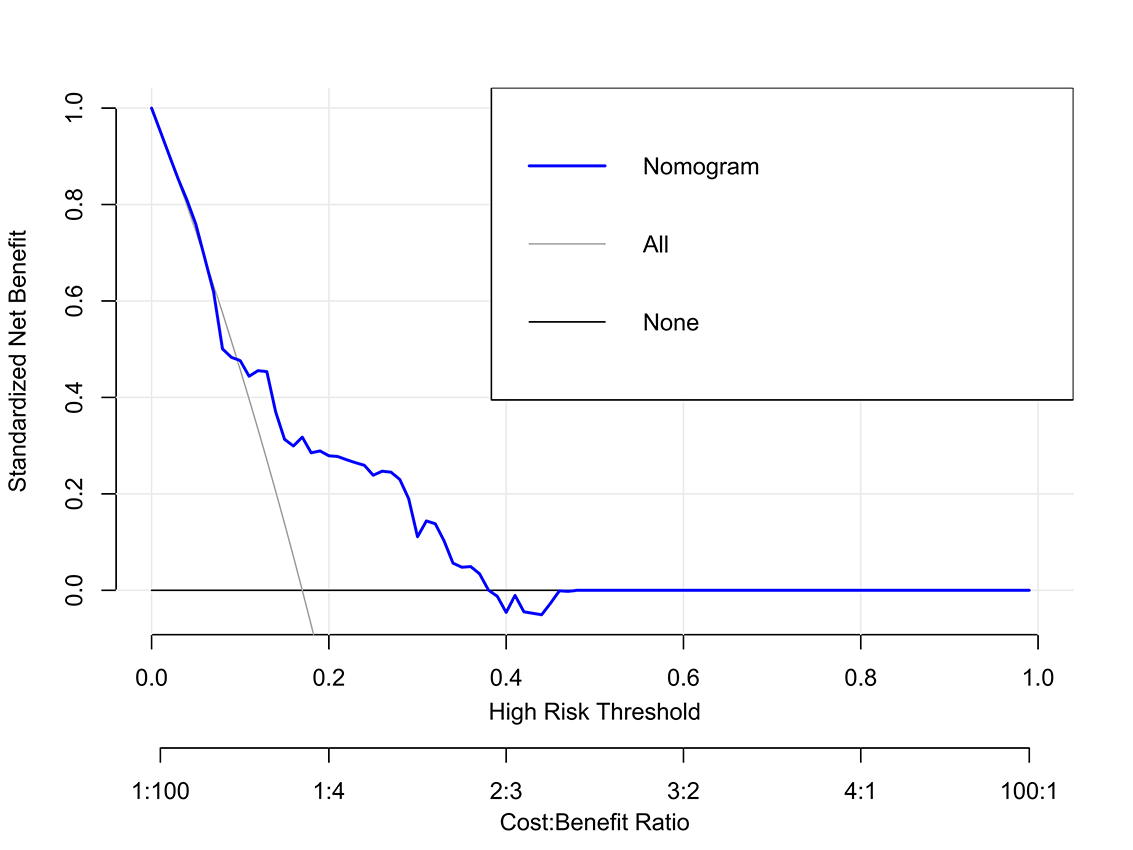
**

**Figure S4 DCA of the nomogram in the external validation set.**

**
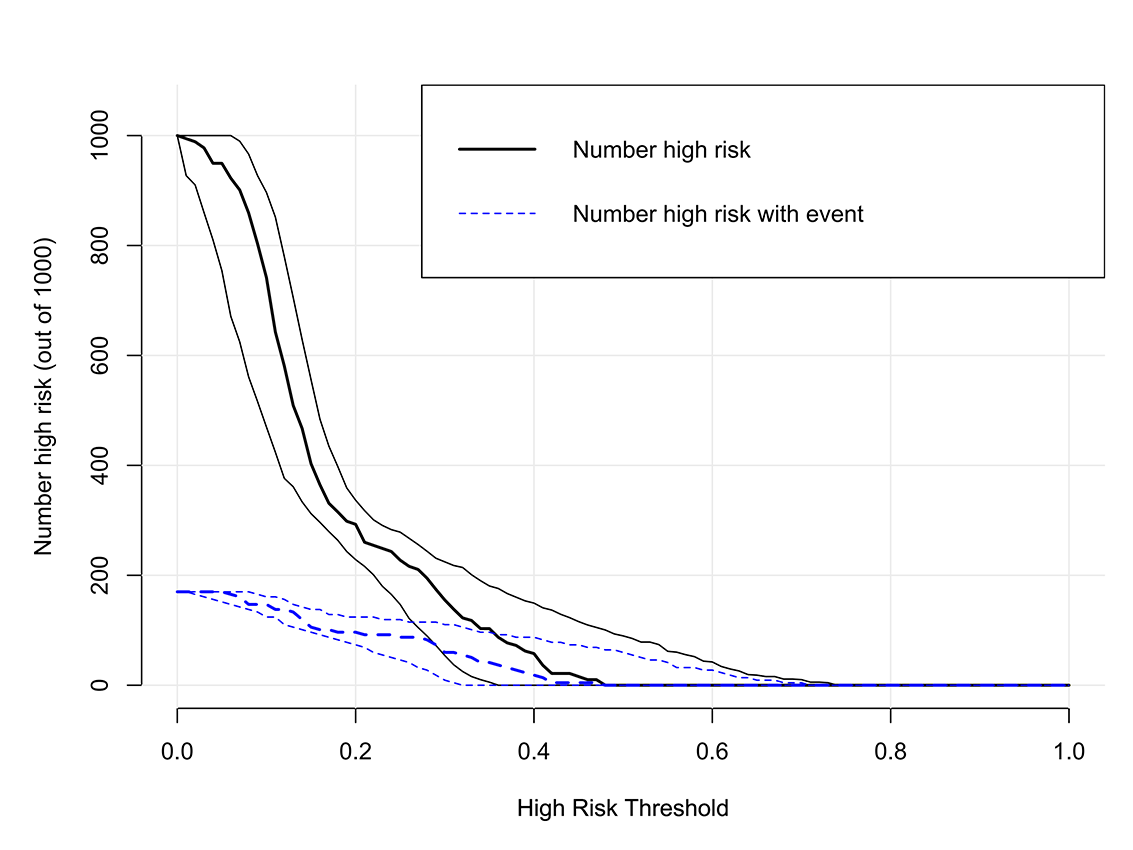
**

**Figure S5 Clinical impact curve of the nomogram in the external validation set.**
